# Center of pressure displacements during gait initiation in individuals with obesity

**DOI:** 10.1186/1743-0003-11-82

**Published:** 2014-05-07

**Authors:** Nicola Cau, Veronica Cimolin, Manuela Galli, Helmer Precilios, Elena Tacchini, Cristina Santovito, Paolo Capodaglio

**Affiliations:** 1 Department of Electronics, Information and Bioengineering, Politecnico di Milano, p.za Leonardo da Vinci 32, 20133 Milan, Italy; 2 IRCCS “San Raffaele Pisana”, Tosinvest Sanità, Rome, Italy; 3 Orthopaedic Rehabilitation Unit and Clinical Lab for Gait Analysis and Posture, Ospedale San Giuseppe, Istituto Auxologico Italiano, IRCCS, Piancavallo (Verbania), Italy

**Keywords:** Gait initiation, Obesity, Rehabilitation, Center of pressure, Anticipatory postural adjustments

## Abstract

**Background:**

Obesity is known to affect balance and gait pattern increasing the risk of fall and injury as compared to the lean population. Such risk is particularly high during postural transitions. Gait initiation (GI) is a transient procedure between static upright posture and steady-state locomotion, which includes anticipatory antero-posterior and lateral movements. GI requires propulsion and balance control. The aim of this study was to characterise quantitatively the strategy of obese subjects during GI using parameters obtained by the Center of Pressure (CoP) track.

**Methods:**

20 obese individuals and 15 age-matched healthy subjects were tested using a force platform during the initiation trials. CoP plots were divided in different phases, which identified the anticipatory postural adjustments (APA_1_, APA_2_) and a movement phase (LOC). Duration, length and velocity of the CoP trace in these phases were calculated and compared.

**Results and discussion:**

The results show that the main characteristic of GI in obese participants is represented by a higher excursion in medio-lateral direction. This condition lead to longer APA length and duration, which are statistical significant during APA2 when compared to control subjects. We also found longer duration of APA1 and LOC phases. In terms of velocity, most of the phases were characterised by a reduced CoP velocity in antero-posterior direction and faster movement in medio-lateral direction as compared to the control group.

**Conclusions:**

Our findings provide novel evidence in GI in obese subjects that may serve for developing exercise programs aimed at specifically improving balance in both the antero-posterior and lateral directions. Such programs together with weight management may be beneficial for improving stability during postural transitions and reducing risk of fall in this population.

## Background

The common act of taking a step involves complex interactions between neural and biomechanical factors that serve to move the body from a quasi-static (quiet standing) to a dynamic state (walking). The biomechanical requirements for successful gait initiation are the generation of a momentum (in the forward direction and in the direction of the stance limb) and the maintenance of balance.

Most studies concerning human gait have focused on steady state walking. However, for safe independent locomotion other aspects of gait are important as well. The transition from standing to walking is a task, which is often required in daily life balance control. Compared to steady state walking, the demands placed on the neuromuscular system are increased in gait initiation, since a complex integration on neural mechanisms, muscle activity and biomechanical forces is necessary [1,2]. Postural adjustment and muscle activity at ankle and hip level are needed to initiate gait.

Gait initiation (GI) is a transient procedure between static upright posture and steady-state locomotion, which includes anticipatory antero-posterior and lateral movements. In addition, it represents the passage from bi- to mono-pedal stance, one of the first phases of the locomotion sequence. The transition from two- to one-foot support also constitutes the first stage in other motor tasks involving lower limbs’ activity.

GI is typically associated with anticipatory postural adjustments (APAs) and occurred prior to gross segmental movement and stability-boundary changes of the first step. APAs mean that the onset of postural changes occurs prior to the onset of the postural disturbance due to the movement. They also mean that a feed-forward postural control is associated with the movement control that prevents the occurrence of movement-related posture and equilibrium disturbances. Specifically, the control of equilibrium during leg movements is interesting to analyse because the moving limb is involved in body support. Movements of the leg change the support conditions and entail a shift of the centre of gravity position prior to movement onset [3].

According to Remelius [4], a postural model considering the shift of COP (Centre of Pressure) and movement of CoM (Centre of Mass) as the result of coordination of the following two mechanisms can be used: an inverted pendulum operating as an “ankle strategy” in the anterior-posterior (A/P) direction, dominated by ankle muscles, and bilateral limb loading creating a “hip strategy” in the medio-lateral (M/L) direction, dominated by hip abductor/adductor muscles [5].

Coordinated actions of these two postural mechanisms move the CoP during APA in the following manner: firstly, a transition of the CoP in lateral and posterior directions together toward the swing foot heel (APA_1_), which is thought to be pre-programmed and requires the bilateral inhibition of soleus and activation of tibialis anterior; secondly, a lateral CoP shift toward the stance foot (APA_2_) [1,4]. The swing leg’s heel-off occurs at the start of the second phase of CoP displacement (a lateral shift toward stance leg) and toe-off (TO) occur just before the forward CoP displacement [6,7]. These APAs reduce the load on the swing leg and are necessary for forward progression [8,9]. Although APAs are referred to as “anticipatory” [10], they also continue throughout the movement itself [5].

GI requires two skills: propulsion and balance control. In particular, the duration of APA is considered an indicator of balance control ability during GI [11]. The skill to maintain stability, weight transfer, foot clearance, etc., become more critical during GI than during the steady state conditions. It was demonstrated that the relative timing of the two previous phases of GI remained consistent at different speed of initiation and stance of tibialis anterior duration during the first phase is inversely related to stance of soleus duration during the second phase [12]. In addition, GI could be positively influenced by the use of orthoses, which have shown to reduce the duration of GI phase in healthy individuals [13].

Patients with neurological disorders, lower limb orthopaedic complications, older adults with poor stability and gait or obese individuals may cope with more difficulty with GI demands [13]. Previous papers have addressed the effect of obesity on balance [14-17] and gait patterns [18] unveiling the motor abnormalities related to an excessive body mass under static and dynamic conditions: such postural and gait impairments increase the risk of fall and injury in obese subjects as compared to the lean population [14].

Propulsion capacity seems also to be reduced in the presence of excessive body mass [19]. In general, obese subjects show a reduced muscle strength in the lower limbs when normalized to body weight, which could account for reduced performance in motor task involving muscle power such as initiating gait, raising from a chair, climbing stairs [20].

Therefore, investigating whether GI, representing the transition from standing with a wide base of support on the two feet to the actual gait with a narrow base and single support, could be affected by the presence of excessive body mass would provide further insight into the functional limitations related to obesity and possibly generate rehabilitation spin-offs. Such transition movements are indeed intrinsically characterised by an increase in postural instability, which can amplify the risk of fall and injuries [21]. In the literature, GI has been so far evaluated in young healthy, elderly, individual with Parkinson, individual with multiple sclerosis and individuals with lower limb amputation [1,2,4,6,21-29]. To the best of our knowledge, no evidence is available for obese subjects. The aim of this study was therefore to quantitatively characterise the strategy of obese subjects during GI using parameters obtained by Center of Pressure (CoP) track.

## Materials and methods

### Subjects

In this study, we analysed 20 obese individuals (BMI ≥30 kg/m^2^; range: 37–52 kg/m^2^): 13 females (age: 49 ± 13 years, height 1.62 ± 0.08 m; weight: 114.08 ± 17.33 kg; BMI: 43.16 ± 4.18 kg/m^2^) and 7 males (age: 37 ± 12.0 years; height: 1.74 ± 0.11 m; weight: 130 ± 29.8 kg; BMI: 42.79 ± 5.42 kg/m^2^). They were selected among obese individuals admitted to the San Giuseppe Hospital, Istituto Auxologico Italiano, in Piancavallo (Italy) for multidisciplinary rehabilitation and weight reduction programs. Exclusion criteria were the presence of neurological disorders, oculo-vestibular disorders, major musculo-skeletal condition (complicated back, hip and knee pain, flat foot), hip and knee replacements, and arrhythmia.

The control group (CG) consisted of 15 age-matched healthy subjects recruited among the hospital staff: 8 females (age: 27 ± 3 years; height: 1.66 ± 0.06 m; weight: 56.7 ± 7 kg; BMI: 20.7 ± 1.2 kg/m^2^) and 7 males (age: 30 ± 5 years; height: 1.80 ± 0.07 m; weight: 71 ± 6 kg; BMI: 21.9 ± 1.2 kg/m^2^).

The characteristics of both groups of participants are reported in Table 1.

**Table 1:** Baseline characteristics of both groups of participants

	**Control Group**	**Obese**
**N(Male/Female)**	15(8/9)	20(7/13)
**Age [years]**	28(4)	45(14)
**Weight [Kg]**	63.4(9.6)	120.2(22.9)*
**High [m]**	1.72(0.1)	1.67(0.1)
**BMI [kg/m**^**2**^**]**	21.2(1.3)	43.0(4.9)*

Note: Values (except N) are shown as mean (SD). *= p < 0.05, obese vs. control group.

N = Number of subjects, BMI = Body Mass Index.

The study was approved by the Ethics Committee of the Istituto Auxologico Italiano; participants were properly informed about aims of the research, testing procedures and personal data treatment. Written informed consent was obtained from each subject before taking part to the experiment.

### Experimental setup

The study was performed in the motion analysis laboratory. Data from the force platform were acquired by means two force platforms (Kistler, Winterthur, Switzerland) in order to measure the trajectory of CoP. Subjects were asked to stand barefoot on first force platform in a relaxed posture, on both legs, in a fixed and parallel position (the distance between their heels was fixed to the pelvis width). Acquisition of force platform data was triggered just prior to the participants receiving a verbal cue to begin walking- approximately 3 seconds before starting task. All the requests were standardized: 3 trials starting with the left leg and 3 trials starting with the right leg. In response to the cue, the participants initiated walking and continued walking for several steps passing through the second platform. For each subject three data collection trials for each limb were performed at a self-selected pace. Thus, a total of 6 gait initiation trials (three starting with the left foot and three with the right foot) were acquired for each participant – both obese and control group.

### Data analysis

The raw COP data sampled at a frequency of 1 kHz and low-pass-filtered at 20 Hz was analysed using a dedicate protocol developed using SmartAnalyzer software (version: 1.10.451.0; BTS, Italy).

**Figure 1:**
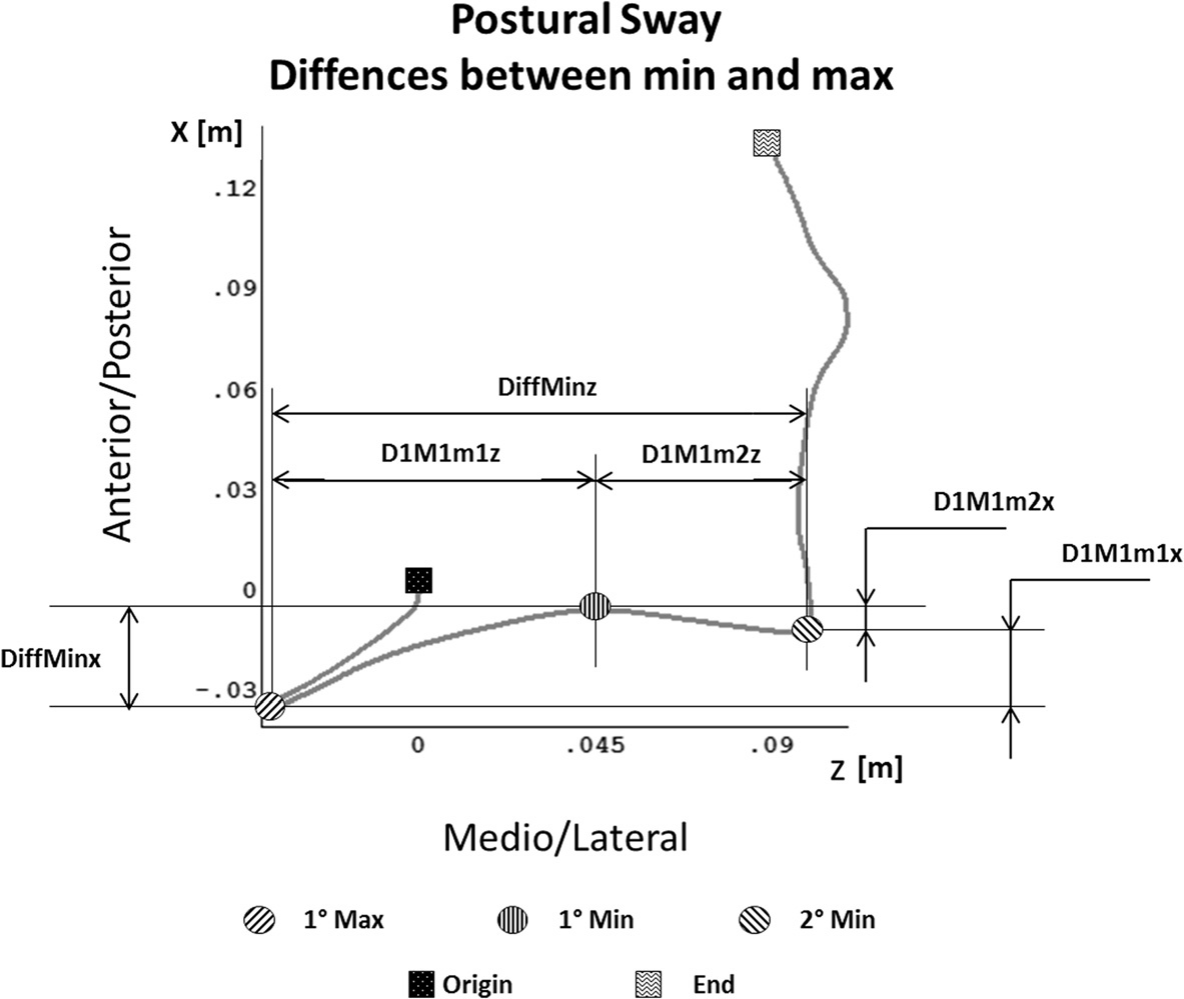
**Overhead view of Centre of Pressure (COP) displacement in antero-posterior and medio-lateral direction during gait initiation (GI).** The initial foot is the left foot. The origin and the end of the GI are marked with squares, minimum and maximum are marked with circle. Other parameter are also indicated; in particular, the differences between minimum and maximum points in antero-posterior and medio-lateral component are shown.

For each acquisition, five points were manually identified as shown in Figure 1:

1. Origin (initial COP position),

2. First minimum (1 min): minimum posterior position of the COP on the leg in swing side,

3. First maximum (1max): Maximum anterior position during the COP transition from the leg in swing to the leg in stance,

4. Second minimum (2 min) minimum posterior position of the COP on the leg in stance side.

5. End (Final COP position)

For the timing analysis, we divided the task in two phases [1,4]:

1) postural phase, which is computed between a quite standing position and the start of the task. This first phase of GI - typically referred to as an APA. It can be divided into two sub-phases:

- APA1 begins at the onset of the movement and ends at the release of swing foot vertical loading – this APA is between the origin and the first minimum. It represents the translation of the CoP in lateral and posterior directions together toward the swing foot heel.

- APA2 that begins at swing foot release, ends at the swing toe off, and represents a lateral CoP shift toward the stance foot [1]. APA2 was further divided into two additional sub-phases: APA2a and APA2b defined respectively the anticipatory movement between the first minimum and the first maximum and the anticipatory movement between the first maximum and the second minimum.

2) locomotor phase - following referred to as LOC – which is between the second minimum and the end of the COP trajectory.

**Figure 2:**
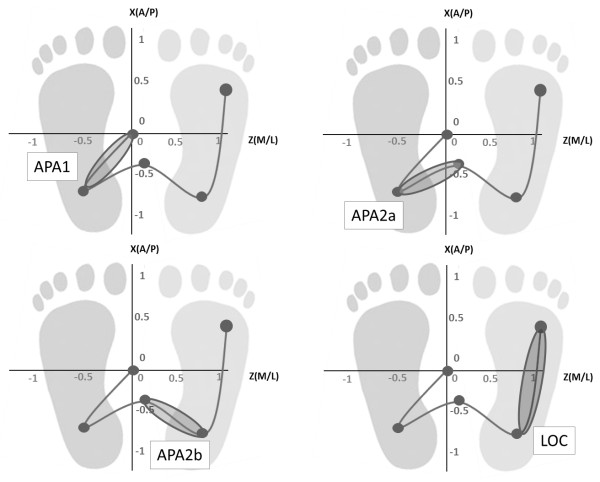
**Centre of Pressure (COP) trajectory division for the analysis (APA1, APA2a, APA2b, LOC).** The initial foot is the right foot.

APA phases and the LOC phase are schematically represented in Figure 2.

According to this phases subdivision, the following parameters have been calculated:

•Track length (m) of the segments: lAPA1, lAPA2a, lAPA2b, lLOC

•Track velocity (m/s) in the segments and in the anterior-posterior direction (x) and in medio lateral direction (z): VAPA1(x,z), VAPA2a(x,z), VAPA2b(x,z), VLOC(x,z)

•Track duration (s) during the segments: dAPA1, dAPA2a, dAPA2b, dLOC and dTot (defined as the whole duration of the track: dAPA1 + dAPA2a + dAPA2b + dLOC).

The relative distance between minimum and maximum have been calculated as shown in Figure 1 and the following parameters were defined:

•DiffMinx defined as the relative distance between first minimum and the second minimum along the anterior-posterior direction-1°Min and 2°Min A/P

•DiffMinz defined as the relative distance between the first minimum and the second minimum along the medio-lateral direction.

•D1M1m1x defined as the relative distance between the first maximum and the first minimum along the anterior-posterior direction-1°Min and 2°Min A/P

•D1M1m1z defined as the relative distance between the first maximum and the first minimum along the medio-lateral direction.

•D1M1m2x defined as the relative distance between the first maximum and the second minimum along the anterior-posterior direction-1°Min and 2°Min A/P

•D1M1m2z defined as the relative distance between the first maximum and the second minimum along the medio-lateral direction.

### Statistical analysis

All parameters were computed bilaterally for each participant and the median and quartile range values of all indexes were calculated for each group (obese and control group). Kolmogorov–Smirnov tests were used to verify if the parameters were normally distributed; the parameters were not normally distributed, so we used the Mann–Whitney U tests for comparing data of obese group and CG. The correlation Statistical significance was set at p < 0.05.

## Results

All the participants were able to complete the instrumented evaluations.

**Figure 3:**
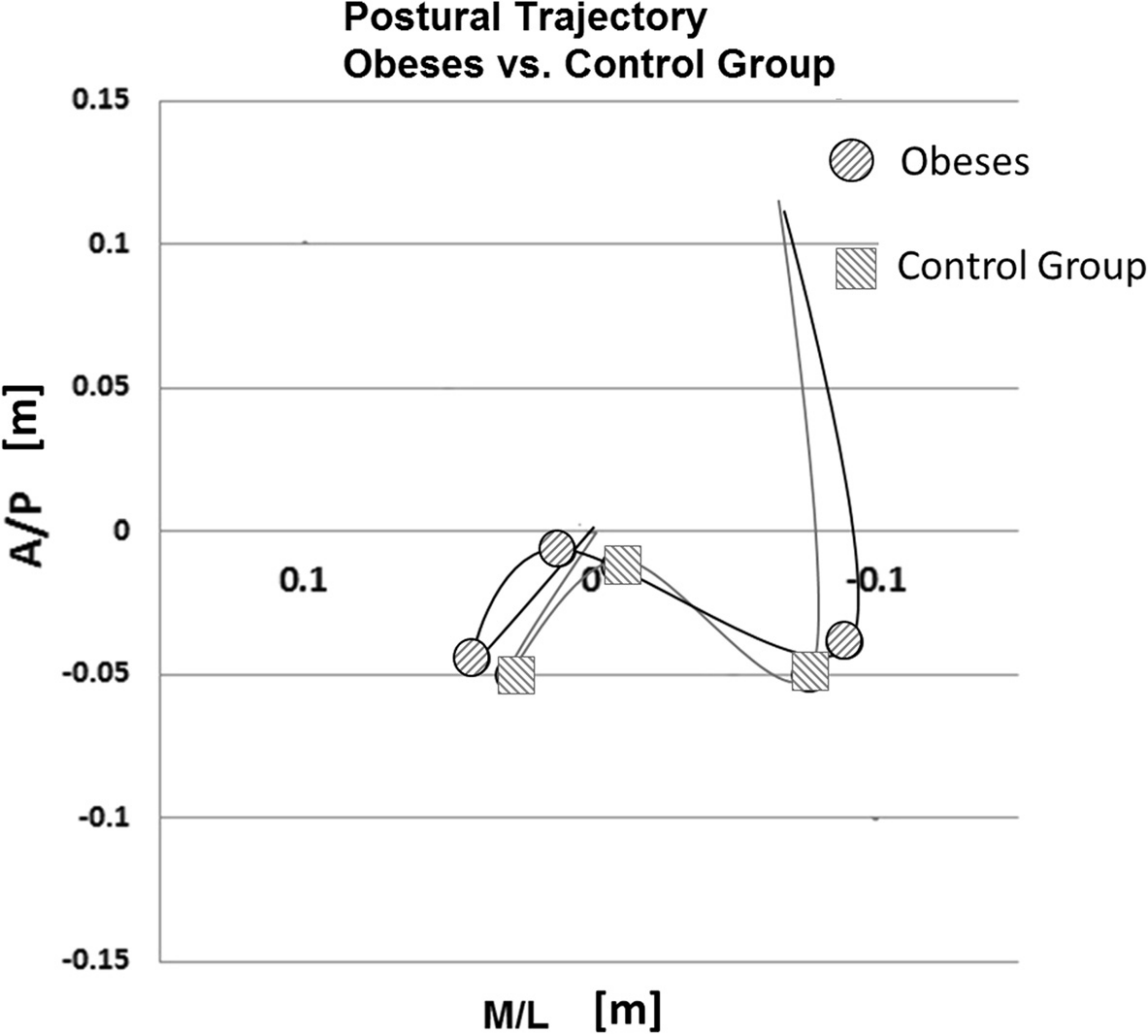
**Postural trajectory comparison between obese and control group.** Minimum and maximum points are marked with circles for obese and with squares for the control group.

Focusing our attention on the trajectory of COP during the acquisition, we have qualitatively observed that no differences were present in term of COP pattern between obese and control group. The same anticipatory postural adjustment strategies were adopted, but the average CoP trajectory of obese subjects seems to be wider as compared to normal-weight subjects (Figure 3).

In Table 2, we have summarised the values of the parameters analysed for the obese and the control group.

**Table 2:** Main parameters

	**Parameters**	**Obese**	**Control Group**
***APAs length***	**lAPA1 [m]**	0.38(0.06)*	0.37(0.05)
	**lAPA2a [m]**	0.08(0.03)*	0. 06(0.02)
	**lAPA2b [m]**	0.05(0.03)	0.05(0.02)
	**lLOC [m]**	0.16(0.04)	0.15(0.02)
***APAs duration***	**dAPA1[s]**	0.32(0.09)*	0.28(0.07)
	**dAPA2a [s]**	0.18(0.07)*	0.15(0.05)
	**dAPA2b [s]**	0.11(0.06)	0.10(0.03)
	**dLOC [s]**	0.59(0.08)*	0.53(0.05)
	**dTot [s]**	1.20(0.14)*	1.06(0.10)
***Maximum and minimum differences***	**D1M1m2x [m]**	0.01(0.01)*	0.02(0.02)
	**D1M1m2z [m]**	0.05(0.02)	0.04(0.02)
	**DiffMinx [m]**	0.02(0.01)	0.01(0.01)
	**DiffMinz [m]**	0.13(0.03)*	0.10(0.02)
	**D1M1m1x [m]**	0.02(0.01)	0.02(0.01)
	**D1M1m1z [m]**	0.08(0.03)*	0.06(0.02)
***APAs velocity***	**V APA1 x [m/s]**	−0.14(0.06)*	−0.19(0.08)
	**V APA1 z [m/s]**	0.13(0.07)*	0.11(0.07)
	**V APA2a x [m/s]**	0.13(0.08)*	0.16(0.08)
	**V APA2a z [m/s]**	0.49(0.23)*	0.40(0.17)
	**V APA2b x [m/s]**	−0.15(0.10)*	−0.20(0.11)
	**V APA2b z [m/s]**	0.47(0.19)	0.46(0.23)
	**V LOC x [m/s]**	0.28(0.08)*	0.29(0.06)
	**V LOC z [m/s]**	0.04(0.03)*	0.02(0.02)

Values of median and quartile range for calculated parameters for obese and control group. *= p < 0.05, obese vs. control group.

As for the APAs length parameters, the lAPA and lAPA2a parameters were statistically different between the two groups and the obese group was characterized by higher values than the control group (Table 2).

In terms of APAs durations, in obese participants the total duration was statistically different and, in particular, APA1, APA2a and LOC resulted the longest phases (dAPA1, dAPA2a and dLOC indices).

As for the parameters related to differences between maximum and minimum, significant differences were found for the relative distance between the first maximum and the second minimum along the anterior-posterior direction (D1M1m2x index), the relative distance between the first minimum and the second minimum along the medio-lateral direction (DiffMinz index) and the relative distance between the first maximum and the first minimum along the medio-lateral direction (D1M1m1z index). In particular, while the value on the antero-posterior direction (D1M1m2x index) in obese group was reduced, on the contrary, the values on the medio-lateral direction (DiffMinz and D1M1m1z indices) were higher in obese as compared to controls.

The parameters related to velocity were significantly different in obese and normal-weight participants. While the velocity on antero-posterior direction (x direction) were lower in the obese group, in the medio-lateral direction (z direction) velocities were higher in obese individuals when compared with normal-weight participants.

## Discussion

The purpose of this study was to investigate the effect of obesity on gait initiation (GI) by quantitatively characterising the spatial-temporal patterns across the GI sequence. Based on our results, the main characteristic of GI in obese participants is represented by a higher excursion in medio-lateral direction, mainly in APA2a and APA2b, as shown by the parameters related to maximum and minimum differences calculated in these two phases. This is in line with previous postural studies on obese subjects [14]. These conditions led to longer APA length and duration, which are statistical significant during APA2a, as compared to control subjects. In addition, we found longer duration of APA1 and LOC phases. These results are in agreement with the visual analysis of the CoP displacements, showing wider CoP trajectories in obese when compared to the controls (Figure 3).

It is noteworthy that crucial abnormalities are present in APA1 and, particularly, in APA2a. These phases of GI represent the APAs starting phases directly connected with the translation of the CoP in lateral and posterior directions together toward the swing foot heel (APA1) and the anticipatory movement from bi- to mono-pedal support with the lateral CoP shift toward the stance foot (APA2a) [4].

As for the LOC phase, we found longer duration in obese than in normal-weight individuals. This could be explained by a possible functional adaptation aimed at improving stability, which is known to be reduced in these individuals [14]. In addition, significant results were obtained with regard to APAs velocity. All of the phases were characterised by a reduced CoP velocity in antero-posterior direction and faster movement in medio-lateral direction as compared to the control group, with the exception of the APA2b phase, where velocity in medio-lateral direction is close to normal. The following factors, together with the maximum and minimum difference parameters, might possibly account for these results: 1) the higher body mass of these individuals, which has already shown to be related to higher displacements in the medio-lateral direction [14]; 2) the continuous balancing effort counteracting the relative instability of those subjects that would ultimately reduce the velocity in antero-posterior direction.

In other pathological conditions, such as Parkinson’s disease and Multiple sclerosis, GI alterations are likely to be directly related to lesions in cerebral areas that are involved in motor programming. Our data cannot be directly compared with similar studies on obese populations in the literature. However, it has been shown that higher body mass induces *per se* a certain degree of instability [14], which is also dependent on fat distribution, with the android fat distribution affecting balance more than the gynoid one [14]. Also, obese subjects have been shown to yield relatively lower muscle strength that their lean counterparts [20]. A reduced capacity of the lower limb distal and proximal muscles in stabilizing the ankle, knee and hip joints of an over-weight body frame while standing or walking may well translate into increased postural sways and decreased dynamic balance [20].

To our knowledge, this is the first study focusing on GI performance in obese subjects and direct comparison with previous researches on this topic cannot be performed. Our results show that the GI pattern is significantly affected by obesity. In particular, the alterations are evident in the early phases of the movement and in medio-lateral direction.

Regarding velocity, it is known that the backward shift of the COP during the postural phase serves to propel the Center of Mass (COM) forward and to reach the intended gait velocity at the end of the first step, which is the LOC phase [8,29-34]. By modulating the velocity of the COM at the end of the first step, these APAs prior to stepping create the optimal postural and dynamic conditions for reaching an adequate progression velocity. Thus, the reduction of the peak of COP backward shift could be a strategy aiming at decreasing the forward propulsion of the COM (i.e., the forward disequilibrium) in order to assure a safe GI movement. This reduction of the progression velocity during GI could possibly explain the reduction in the COP velocity during the LOC phase.

Our findings could be interesting from a rehabilitative point of view as they provide evidence for developing exercise programs aimed at specifically improving balance in both the antero-posterior and in particular in lateral directions. Comprehensive rehabilitation programs including weight management and tailored strengthening and balance exercises may be beneficial in fact for improving stability during postural transitions and reducing risk of fall in this population.

The present study have some limitations. First, only data related to CoP trajectory were investigated while no evaluations of lower limb joints kinematics and kinetics were conducted. However, as it represents the first attempt to quantify GI strategy of obese individuals, we decided to perform the GI assessment using only one force platform, with a less time-consuming evaluation than including markers placement or other conventional assessments. Future research could address the quantification of GI strategy including a stratification of participants based on BMI, in order to investigate whether BMI influences GI performance.

## Conclusions

Our study outlines the limitations of obese individuals during GI performance. Such limitations are especially evident in the early phases of the movement and in medio-lateral direction. The GI assessment could integrate the functional assessment performed on obese subjects. The GI evaluation test has the advantage to be simple and less time-consuming than other conventional tests, such standard gait analysis and it requires only one force platform, which can be fitted in an out-patients department.

## Competing interests

All authors haven’t any conflicts of interest and any financial interest. All authors attest and affirm that the material within has not been and will not be submitted for publication elsewhere.

## Authors’ contributions

NC made substantial contributions to data elaboration and analysis and was involved in drafting the manuscript. VC made substantial contributions to analysis and interpretation of data and was involved in drafting the manuscript. MG made contribution to conception, design and interpretation of data, revising the manuscript critically and gave the final approval of the manuscript. HP made contributions to data acquisition. ET made contribution to participants’ selection. CS made contribution to data acquisition. PC made contribution to conception, design and interpretation of data, revising the manuscript critically and gave the final approval of the manuscript. All authors read and approved the final manuscript.
